# Detection and Characterization of Gastric Cancer Using Cascade Deep Learning Model in Endoscopic Images

**DOI:** 10.3390/diagnostics12081996

**Published:** 2022-08-18

**Authors:** Atsushi Teramoto, Tomoyuki Shibata, Hyuga Yamada, Yoshiki Hirooka, Kuniaki Saito, Hiroshi Fujita

**Affiliations:** 1 School of Medical Sciences, Fujita Health University, Toyoake 470-1192, Japan; 2 Department of Gastroenterology and Hepatology, Fujita Health University, Toyoake 470-1192, Japan; 3 Faculty of Engineering, Gifu University, Gifu 501-1194, Japan

**Keywords:** gastric cancer, deep learning, convolutional neural network, segmentation, classification

## Abstract

Endoscopy is widely applied in the examination of gastric cancer. However, extensive knowledge and experience are required, owing to the need to examine the lesion while manipulating the endoscope. Various diagnostic support techniques have been reported for this examination. In our previous study, segmentation of invasive areas of gastric cancer was performed directly from endoscopic images and the detection sensitivity per case was 0.98. This method has challenges of false positives and computational costs because segmentation was applied to all healthy images that were captured during the examination. In this study, we propose a cascaded deep learning model to perform categorization of endoscopic images and identification of the invasive region to solve the above challenges. Endoscopic images are first classified as normal, showing early gastric cancer and showing advanced gastric cancer using a convolutional neural network. Segmentation on the extent of gastric cancer invasion is performed for the images classified as showing cancer using two separate U-Net models. In an experiment, 1208 endoscopic images collected from healthy subjects, 533 images collected from patients with early stage gastric cancer, and 637 images from patients with advanced gastric cancer were used for evaluation. The sensitivity and specificity of the proposed approach in the detection of gastric cancer via image classification were 97.0% and 99.4%, respectively. Furthermore, both detection sensitivity and specificity reached 100% in a case-based evaluation. The extent of invasion was also identified at an acceptable level, suggesting that the proposed method may be considered useful for the classification of endoscopic images and identification of the extent of cancer invasion.

## 1. Introduction

### 1.1. Background

Gastric cancer is a malignant tumor that mainly affects the gastric mucosa and is the second-most-common cause of death among all cancers after lung cancer [1,2]. It is a highly regional disease, with more than 50% of cases occurring in East Asia. Diagnostic and treatment techniques for gastric cancer continue to improve and early detection has been shown to reduce mortality rates among patients with gastric cancer.

Gastric examination is performed by endoscopy and gastrofluoroscopy using barium. Because endoscopy has superior sensitivity in detecting early stage gastric cancer and allows for tissue collection and treatment under observation, it has been widely adopted for gastric cancer screening and detailed diagnosis. In endoscopic diagnosis, a physician checks the endoscopic images while operating the endoscope and records still images at key points when abnormalities are detected. In addition, magnification and staining are often performed to classify diseases and diagnose the extent and depth of lesions [3].

However, the procedure is highly complex, requiring multiple tasks to be performed during the examination. Hence, there is some concern that lesions may be missed. According to the results of a survey, the probability of missing a lesion during endoscopy was 22.2% and the accuracy of diagnosis depends, to a considerable extent, on the experience and skill of the physician. Therefore, technologies to assist examinations are strongly desired to reduce the burden on the physician and to improve diagnostic accuracy.

In recent years, artificial intelligence technologies have made remarkable progress and deep learning technology has shown excellent performance in the field of image recognition [4,5]. Deep learning techniques have been proposed for various applications involving different types of medical images [6,7,8]. In this study, we propose a deep-learning-based method for the detection and identification of the depth and extent of cancer invasion to support endoscopy.

### 1.2. Related Works

Various studies have been conducted on the automated detection of gastric cancer lesions in endoscopic images, the extraction of the extent of invasion, and the evaluation of cancer depth [9,10,11,12,13,14,15]. Among early works on the subject, Hirasawa et al. developed a method for automated detection of early gastric cancer lesions and extraction of the extent of invasion using a single-shot detector (SSD), an object detection model [10]. The sensitivity of detection using 15,880 original endoscopic images was 92.2% and the positive predictive value was 30.6%. Sakai et al. developed a method to detect gastric cancer by feeding a convolutional neural network (CNN) with finely cut patches of endoscopic images to classify areas showing gastric cancer and normal areas [11] and achieved a detection sensitivity and specificity using 926 original images of 80.0% and 94.8%, respectively. Shibata et al. proposed a method for detecting the presence of early gastric cancer and extracting invasive regions using Mask R-CNN, which was designed to perform both object detection and segmentation [12]. They achieved a sensitivity of 96.0% and a segmentation concordance of 71% using 1741 original endoscopic images in the automated detection of early gastric cancer. Teramoto et al. proposed a U-Net R-CNN model to detect early gastric cancer using two CNNs to perform segmentation and classification [13]. First, they used U-Net, a CNN model for segmentation, to detect regions of early gastric cancer. Then, the detected regions were classified as normal or containing gastric cancer by a CNN model. They obtained a detection sensitivity of 98% and reduced false positives by 70% compared to their previous study on the same image database.

To evaluate the depth of gastric cancer, Zhu et al. used a CNN to classify cancer as remaining within the mucosa or as having invaded the submucosa, the latter indicating an advanced cancer. The evaluation resulted in a sensitivity of 76.47% and a specificity of 95.56% using the original 993 endoscopic images [14]. Hamada et al. also proposed a method to classify early gastric cancer as mucosal or submucosal using a CNN and obtained a classification accuracy of 78.9% using 3508 original images [15]. Endoscopic ultrasonography (EUS) is often used to assess the depth and internal structure of upper gastrointestinal lesions, including gastric cancer, and diagnostic assistive technologies are required [16,17]. Hirai et al. used an AI system to classify subepithelial lesions (SEL) using EUS images. The results showed that the correct classification rate of the images was 86.1% using 16,110 original images, which was much higher than that of endoscopists [17].

These techniques present independent methods to detect the presence and evaluate the depth of gastric carcinoma. In our previous study, semantic segmentation was applied directly to endoscopic images; however, the many healthy images collected during the examination were subject to processing, resulting in false positives and high computational cost. The actual detailed diagnoses were performed by first detecting abnormal areas during the endoscopic procedure and approaching the lesion. Hence, a more automated detection and classification scheme in line with the diagnostic flow is desirable. This cascaded processing has been applied to other medical images. For example, Krzysztof et al. proposed a method for segmenting brain tumors from head MR images that uses two types of U-Nets to perform tumor location identification and detailed multiclass segmentation [18]. Jie et al. proposed a cascaded network model for the segmentation of brain regions in 3T head MR images in combination with 7T head MR images and obtained better results than conventional methods [19]. In addition, Nina et al. analyzed the aortic root using a cascade of two types of CNNs [20]. These methods do not use a single network model to perform a single task but divide the roles among multiple networks. Similar to these methods, a performance improvement can be expected in the detection of gastric cancer using endoscopic images by introducing a cascade structure of two different tasks. In this study, we propose a cascade deep learning model designed to perform automatic detection and classification, which combines image classification and region extraction to detect normal and gastric cancer, evaluate the depth of the lesion, and identify the extent of invasion.

The organization of this paper is as follows: In the *Methods*, we propose a cascade-based gastric cancer detection and classification method. In the *Results*, we evaluate the detection characteristics by calculating the detection performance and activation maps. In the *Discussion* section, the detection performance and its comparison with previous studies are discussed. Finally, in the *Conclusions*, we describe the effectiveness and contribution of this study.

## 2. Methods

### 2.1. Proposed Method

**Figure 1:**
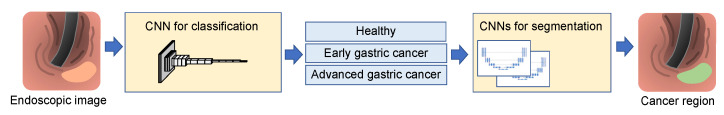
Outline of the proposed method.

Figure 1 shows an outline of the proposed method. Still images taken during the endoscopy procedure are fed to a CNN model trained to perform image classification and are classified as healthy, indicating early gastric cancer, and indicating advanced gastric cancer. Then, the images classified as showing gastric cancer are input into a CNN model trained to perform segmentation to extract the regions of early and advanced gastric cancer and the extent of invasion is identified.

### 2.2. Image Dataset

For this study, endoscopic images were collected from healthy subjects, patients with early stage gastric cancer and patients with advanced gastric cancer. All images were collected at the endoscopy center of Fujita Health University Hospital during an examination. They comprised 1208 endoscopic images collected from 42 healthy subjects, 533 images from 93 patients with early gastric cancer, and 637 images from 50 patients with advanced gastric cancer. Images were obtained using upper endoscopes (GIF-290Z, GIF-HQ290, GIF-XP290N, GIF-260Z; Olympus Medical Systems, Co., Ltd., Tokyo, Japan; and EG-L600ZW7; Fujifilm Corp., Tokyo, Japan) and standard endoscopic video systems (EVIS LUCERA CV-260/CLV-260, EVIS LUCERA ELITE CV-290/CLV-290SL; Olympus Medical Systems; and VP-4450HD/LL-4450; Fujifilm Corp.). The image matrix sizes ranged from 640 × 480 to 1440 × 1080 pixels; these were standard white-light images stored in JPEG format. This study was approved by an institutional review board and patients’ informed consent was obtained under the condition that all data were anonymized (No. HM17-226).

Here, early gastric cancer was defined as lesions that remained in the mucosal or submucosal layer of the stomach, whereas advanced gastric cancer was defined as lesions that reached to the intrinsic muscular layer or deeper than that layer. For healthy subjects, we reassessed the cases diagnosed as normal by the endoscopists and characterized a case as “healthy” when there was no specific lesion, such as a polyp, tumor, or gastritis.

**Figure 2:**
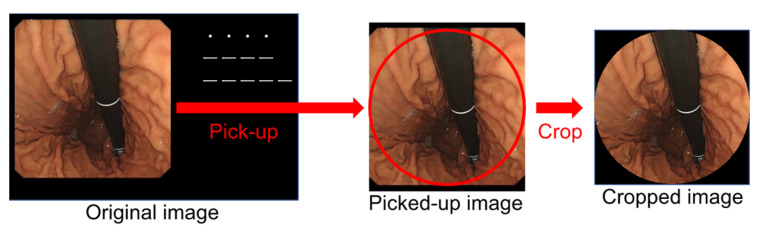
Image cropping for regularization.

**Figure 3:**
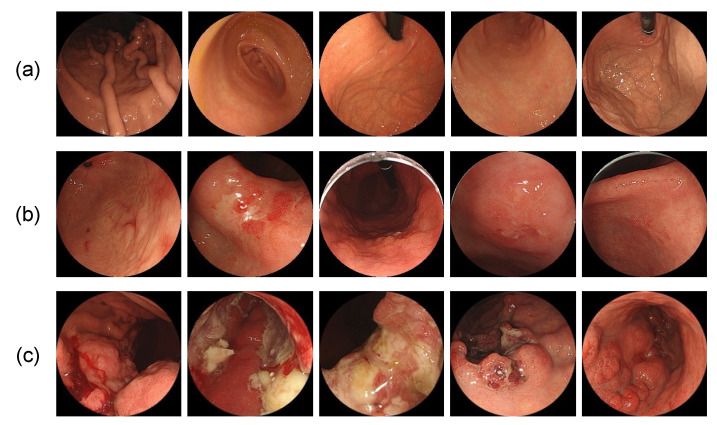
Sample images of the image dataset. (**a**) healthy subjects; (**b**) early gastric cancer; (**c**) advanced gastric cancer.

Images stored by endoscope systems may contain text information, such as patient names, and the shape of the field of view differs depending on the model of the endoscope. Therefore, as shown in Figure 2, a perfect circle was inscribed in the effective field of view of the endoscope, the area outside of the circle was filled in black, and a square area was cropped with the bounding rectangle of the circle. The circular field of view was implemented to avoid the bias caused by different endoscope models and maintain image uniformity when data augmentation was performed as described below. Sample images of the dataset are shown in Figure 3.

### 2.3. Annotation of Gastric Cancer Region

For images diagnosed as gastric cancer, labeled images of gastric cancer areas were created for the segmentation of gastric cancer areas. Using an in-house annotation tool, labeled images with the invasion areas of gastric cancer marked were created and confirmed by a board-certified endoscopist (T. S.).

### 2.4. Data Augmentation

The number of images in the three categories differed. In addition, training a network using a small number of images taken at a certain angle of view may result in overfitting. In endoscopic examination, images of a single lesion are taken from various positions and distances and there is wide variation in the position and size of the lesion in the image, with sufficient data available. On the other hand, there is not much variation in the rotation of images due to the limitations of examination techniques. Therefore, to avoid bias and overfitting caused by the number of images and viewing angle, data augmentation was performed by image rotation [12]. The rate of augmentation was varied according to the number of original images belonging to the class. Images of healthy subjects were augmented by a factor of 4 at a pitch of 90 degrees, images of early gastric cancer were augmented by a factor of 9 at a pitch of 40 degrees, and images of advanced gastric cancer were augmented by a factor of 7 at a pitch of 50 degrees.

### 2.5. Network Architecture for Image Classification

The proposed method involves image classification and segmentation by two CNNs, as shown in Figure 1. First, the given images are classified as healthy, showing early gastric cancer or showing advanced gastric cancer by a CNN model. In this study, VGG-16/19 [21], InceptionV3 [22], ResNet-50/-101/-152 [23], and DenseNet-121/-169/-201 [24] models were constructed and compared in terms of their classification performance.

We introduced transfer learning to the training of the CNN described above, which involves transferring the processing ability acquired in solving one task to learning to solve another task. If the CNN of the original diversion is a well-trained model with a huge amount of data, so it may provide a higher capacity [25]. In this study, a CNN model trained on the ImageNet database consisting of more than 10 million images was adapted for classification of endoscopic images. To conduct the transfer learning, the fully connected layers of the original CNNs were removed, 1024 and 2 units of fully connected layers were newly connected, and the weights of the fully connected layers were adjusted by training using endoscopic images. We used the Adam optimizer with a learning rate of 0.0001 and 50 training epochs.

### 2.6. Network Architecture for Image Segmentation

Images classified by the first-stage CNN were transferred to the next-stage CNN for segmentation according to their classification results. Images classified as healthy were judged as not including cancer areas and, thus, segmentation was not performed. Images classified as showing early and advanced gastric cancer were fed to a dedicated CNN for segmentation processing.

**Figure 4:**
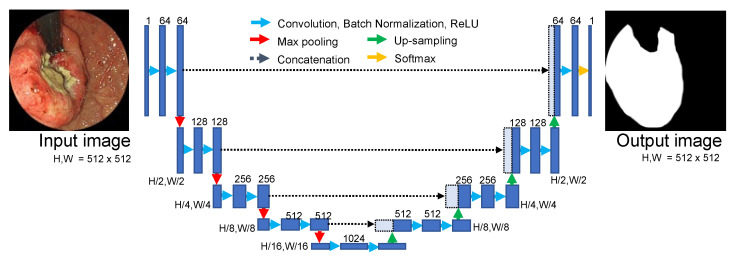
U-Net architecture for gastric cancer segmentation.

We implemented U-Net as a CNN model to perform classification, given its excellent performance in medical image segmentation [26]. The structure of the network is shown in Figure 4 and included five layers of encoders and decoders. The encoders extract the features of the image. The decoders upscale the extracted feature maps and generate a pattern similar to that of the label image given as the teaching data. Furthermore, between the same layer of encoders and decoders, spatial information reduced by the encoder layer is given to the decoders by a mechanism called skip connections, which transfer information between the encoder and decoder layers.

Here, the morphology shown in images of early and advanced gastric cancer differs considerably. Therefore, two independent U-Nets were created to segment early and advanced gastric cancer. The Adam optimizer was used for training with a learning coefficient of 0.0001 and 50 training epochs.

### 2.7. Evaluation Metrics

To confirm the effectiveness of the proposed method, we evaluated the output of the CNN models for image classification and segmentation. First, to evaluate the performance of the CNN for image classification in the first stage, a confusion matrix was created based on the CNN classification results. Based on the matrix, we calculated the accuracy, sensitivity, and specificity of the models. N_Healthy_, N_EGC_, and N_AGC_ indicate the numbers of images for healthy, early gastric cancer, and advanced gastric cancer, respectively, and C_Healty_, C_EGC_, and C_AGC_ are the numbers of images successfully classified as healthy, early gastric cancer, and advanced gastric cancer, respectively.

Accuracy_Overall_ = (C_Healty_ + C_EGC_ + C_AGC_)/(N_Healthy_ + N_EGC_ + N_AGC_) (1)

Accuracy_Health_ = C_Healty_/N_Healthy_ (2)

Accuracy_EGC_ = C_EGC_/N_EGC_ (3)

Accuracy_AGC_ = C_AGC_/N_AGC_ (4)

Accuracy_Balanced_ = (Accuracy_Health_ + Accuracy_EGC_ + Accuracy_AGC_)/3 (5)

Sensitivity = (C_EGC_ + C_AGC_)/(N_EGC_ + N_AGC_) (6)

Specificity = C_Healty_/N_Healthy_ (7)

The above indices were evaluated on an image-by-image basis (image-based evaluation) and on a case-by-case basis (case-based evaluation). For the former, the results were tabulated when each image was classified into the class with the largest CNN output value. For the case-by-case evaluation, the output values of images collected from the same case were averaged for each class and the class with the highest average value was considered as the classification result.

As for the visualization method of CNN output, class activation mapping (CAM) calculates what parts of an image influence the prediction based on the feature map during inference. Among various CAM methods, Grad-CAM calculates the activation map by calculating the gradient of the CNN feature map and can provide a stable activation map regardless of the model [27]. In this study, we calculated activation maps for healthy cases, early gastric cancer, and advanced gastric cancer using Grad-CAM to visualize the basis for classification.

In the second step of the segmentation process, the Dice coefficient (Di) and Jaccard coefficient (Ji) were used to evaluate the extent to which the invasive area output by U-Net was correctly extracted. Di and Ji were defined by the following equation to evaluate the degree of agreement between the label image output by U-Net and the correct label (Ground Truth) created by the endoscopist.

Di = 2|A∩B|/(|A| + |B|) × 100 [%](8)

Ji = |A∩B|/|A∪B| × 100 [%](9)

where A is the ground truth prepared by the gastroenterologist and B represents the region of gastric cancer obtained by the CNN. Cross-validation was used to evaluate the classification and segmentation process [28]. In cross-validation, the image dataset was divided into K subsets to avoid fragmenting the cases. The CNN was trained on K-1 subsets and the image data belonging to the remaining one subset was defined as testing data; then, the classification results were evaluated. In the cross-validation method, the test results for all data were obtained by training and testing K times while changing the subset used as the testing data. The data were divided into 5 subsets (5-fold cross-validation) and the classification performance was evaluated.

The calculations of two CNNs were performed using software we developed in the Python programming language with an AMD Ryzen 9 3950X processor (16 CPU cores, 4.7 GHz) with 128 GB of DDR4 memory. The training processes of the CNNs were accelerated by an NVIDIA Quadro RTX 8000 GPU (48 GB memory).

## 3. Results

### Image Classification Result

**Figure 5:**
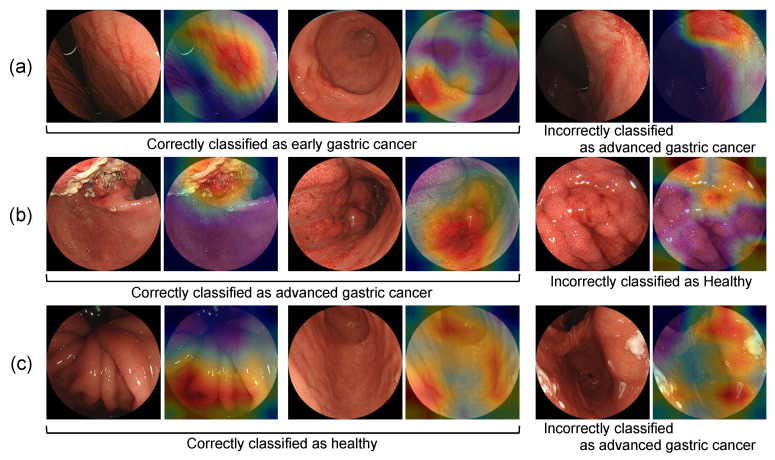
Correctly and incorrectly classified images: (**a**) classification results of early gastric cancer; (**b**) classification results of advanced gastric cancer; (**c**) classification results of healthy subjects.

Table 1 shows the results of the comparison of the classification performance of the CNN models for image classification, which performed in the first stage of this study. DenseNet-121 had the highest classification performance for both image-based and case-based classification, with a detection sensitivity and specificity of 0.970 and 0.994, respectively, in the image-based evaluation. In the case-based evaluation, all of the values were 1.0. A confusion matrix of the classification results from DenseNet-121 is shown in Table 2. Next, the correctly and incorrectly classified images are shown in Figure 5, along with the Grad-CAM output.

**Figure 6:**
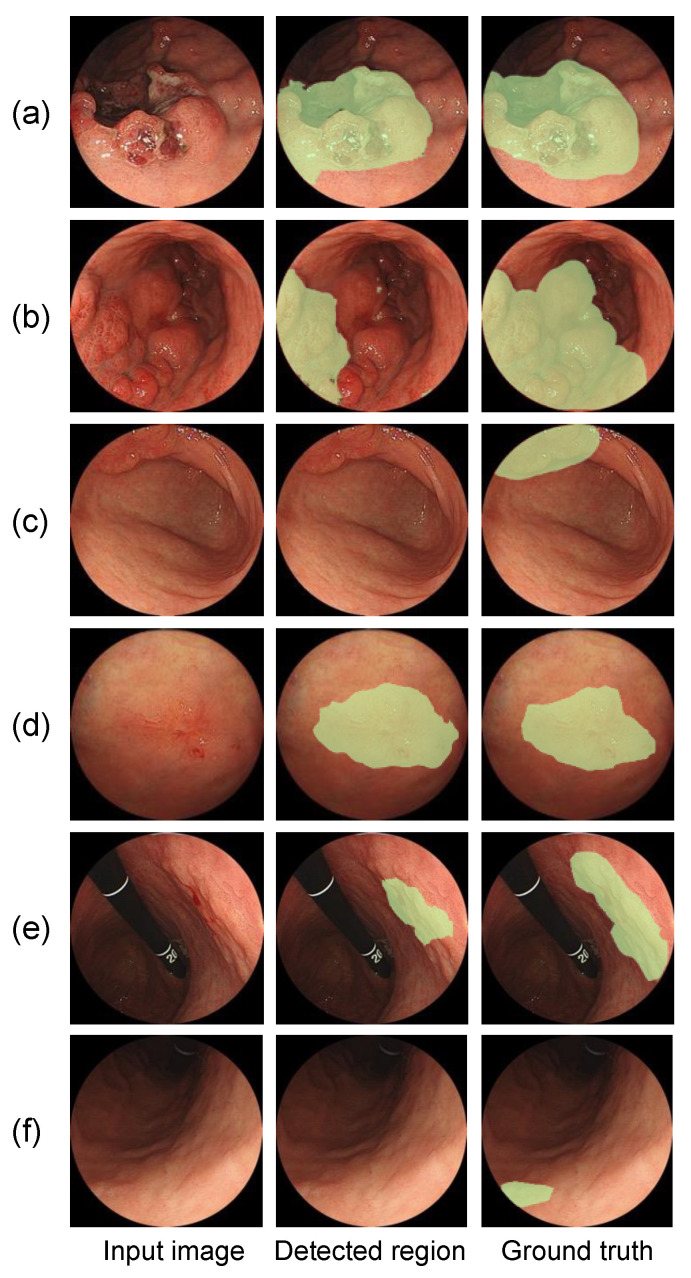
Lesions detected and missed in the segmentation phase: (**a**,**b**) correctly detected region of advanced gastric cancer; (**c**) missed region of advanced gastric cancer; (**d**,**e**) correctly detected region of early gastric cancer; (**f**) missed region of early gastric cancer.

The results of U-Net segmentation are shown in Figure 6 and the Dice and Jaccard coefficients are listed in Table 3. Note that the segmentation of healthy images often extracted false-positive regions. The evaluation results showed that 283 false-positive regions were detected in 1208 healthy images and the number of false positives per image (FPI) was 0.234 (283/1208). Because the proposed method performs image classification in the first stage, images classified as healthy need not be segmented. The false positives from the segmentation results were investigated by excluding the images correctly classified as healthy in the first stage and false-positive regions were extracted from six images, yielding an FPI of 0.005 (6/1208).

## 4. Discussion

In this study, we proposed a cascaded deep learning model for endoscopic images, designed to classify the depth of normal and gastric cancer using a CNN model to perform image classification and then performed segmentation on the extent of gastric cancer invasion using U-Net.

In the first stage of the image classification task, nine different CNN models were employed and their classification performances were compared. The results showed that the performance differences among the models were not large, being within 5% for most of the evaluation indices. Among the CNN models, DenseNet-121 achieved the highest classification performance for all items, with a detection sensitivity of 97% and a specificity of 99.4% for gastric cancer. A slight misclassification occurred in the image-based evaluation. In cases of early gastric cancer, images with extensive areas of invasion were classified as advanced gastric cancer. In healthy cases, some bubbles adhering to the gastric mucosa and some hyperemic areas produced by endoscopy were misclassified as advanced gastric cancer. Endoscopy involves collecting multiple images for a single patient. The classification results of the individual images were tabulated and a decision was made for each patient. Consequently, all cases were classified into the correct category, as shown in Table 2. These results indicate that the CNN established in the first stage was able to accurately classify not only normal samples and those exhibiting gastric cancer but also the depth of the cancer.

As a result of acquiring the regions of interest for image classification by Grad-CAM, the lesion and its surrounding area tended to be focused on for early and advanced gastric cancer. In healthy subjects, the results showed that the entire gastric mucosa was uniformly focused on. These results are similar to the process of diagnosis using images, which can be considered reasonable.

In the second stage of the cascade model, the invasive extent was segmented by U-Net for images classified as early or advanced gastric cancer and the agreement between the invasive extent output by U-Net and the gold standard specified by the endoscopist was evaluated using the Dice and Jaccard coefficients. The Dice coefficient was 0.555 for early gastric cancer and 0.716 for advanced gastric cancer. This was due to the lack of morphological changes in the images of early gastric cancer and the fact that many of the images were taken from the side of the gastric wall rather than from the front, so the contours of the invasive area were not completely defined. Even with this underestimation, the extent of the invasion output, like the Grad-CAM output, provides a basis for image classification and can provide important information during endoscopy.

To compare the detection performance of gastric cancer, Table 4 shows the performances of previous studies and the proposed method. In our previous study [13], we presented a method that obtained a case-based detection sensitivity of 0.989, and the number of false positives (FPI) per image was 0.011. When compared under the same conditions, those obtained in the present work were 1.000 and 0.005, respectively.

Our previous study faced challenges about false positives and high computational cost because semantic segmentation was applied to all healthy images collected during the examination. The detection sensitivity and the number of false positives in the proposed cascade model were superior to those of our previous study and the computational cost of the segmentation process was eliminated because healthy images were cut off by the classification CNN. Although an accurate comparison is not possible because a different database was used, the proposed method with cascaded model had a better detection sensitivity than those in previous studies using an SSD (sensitivity of 99.2%) [10]. The proposed method can classify images with high sensitivity and specificity and can also provide the extent of invasion, suggesting that it may be considered a highly effective technique for assisting in endoscopic screening of gastric cancer.

A limitation of this study is that the endoscopic images were collected at a single facility. In the future, an evaluation of usefulness of the proposed approach should be conducted using images from multiple facilities. Regarding the evaluation of the extraction accuracy of the invasive area, the Dice and Jaccard coefficients were introduced. However, a subjective evaluation of whether the clinically important invasive area was extracted is required. Furthermore, we intend to develop endoscopy support software that incorporates the constructed model and confirm its usefulness in practical medicine.

## 5. Conclusions

In this study, we proposed a cascade deep learning model to support the automated detection and classification of gastric cancer during endoscopy by combining image classification and segmentation to detect normal samples and gastric cancer, to evaluate the degree of depth, and to identify the extent of invasion. The evaluation results showed that the accuracy of first-stage image classification was high and the proposed method accurately classified normal images and gastric cancer and classified the depth of invasion. In addition, the extent of gastric cancer invasion was correctly extracted from several images. The number of false positives generated by the proposed method was significantly low, indicating that the method is effective for endoscopic screening.

## Figures and Tables

**Table 1:** Comparison of CNN models for image classification. Values in bold indicate the results of the CNN model with the highest performance.

(**a**) **Image-Based Classification**							
	**Accuracy_Healthy_**	**Accuracy_EGC_**	**Accuracy_AGC_**	**Accuracy_overall_**	**Accuracy_Balanced_**	**Sensitivity**	**Specificity**
VGG16	0.964	0.919	0.922	0.943	0.935	0.951	0.964
VGG19	0.994	0.947	0.904	0.960	0.949	0.939	0.994
InceptionV3	0.988	0.921	0.906	0.951	0.938	0.938	0.988
DenseNet121	0.994	0.998	0.945	0.982	0.979	0.970	0.994
DenseNet169	0.995	0.998	0.903	0.971	0.965	0.948	0.995
DenseNet201	0.996	0.989	0.943	0.980	0.976	0.965	0.996
ResNet50	0.991	0.991	0.922	0.972	0.968	0.956	0.991
ResNet101	0.990	0.981	0.920	0.969	0.964	0.949	0.990
ResNet152	0.996	0.994	0.918	0.975	0.970	0.954	0.996
(**b**) **Case-based classification**							
	**Accuracy_Healthy_**	**Accuracy_EGC_**	**Accuracy_AGC_**	**Accuracy_overall_**	**Accuracy_Balanced_**	**Sensitivity**	**Specificity**
VGG16	0.976	0.937	0.960	0.952	0.958	0.979	0.976
VGG19	1.000	0.947	0.940	0.957	0.962	0.972	1.000
InceptionV3	1.000	0.958	0.980	0.973	0.979	0.986	1.000
DenseNet121	1.000	1.000	1.000	1.000	1.000	1.000	1.000
DenseNet169	1.000	1.000	0.960	0.989	0.987	0.986	1.000
DenseNet201	1.000	1.000	0.980	0.995	0.993	0.993	1.000
ResNet50	1.000	0.989	1.000	0.995	0.996	1.000	1.000
ResNet101	1.000	1.000	0.980	0.995	0.993	0.993	1.000
ResNet152	1.000	1.000	0.980	0.995	0.993	0.993	1.000

**Table 2:** Confusion matrices of classification using DenseNet121.

(**a**) **Image-Based Classification**				
		**Predicted**		
		**Healthy**	**Early Gastric Cancer**	**Advanced Gastric Cancer**
Actual	Healthy	1201	0	7
	Early gastric cancer	0	531	1
	Advanced gastric cancer	35	0	602
(**b**) **Case-based classification**				
		**Predicted**		
		**Healthy**	**Early Gastric Cancer**	**Advanced Gastric Cancer**
Actual	Healthy	42	0	0
	Early gastric cancer	0	95	0
	Advanced gastric cancer	0	0	50

**Table 3:** Evaluation results of cancer segmentation.

	Di	Ji
Early gastric cancer	0.555	0.427
Advanced gastric cancer	0.716	0.611

**Table 4:** Performance comparison of gastric cancer detection and segmentation.

Author	Method	Image Dataset	Detection Performance	Segmentation Performance
Hirasawa et al. [10]	SSD	Original 15,880 images	Sensitivity = 0.922 Positive predictive value = 0.306	-
Sakai et al. [11]	CNN	Original 926 images	Sensitivity = 0.800 Specificity = 0.948	-
Shibata et al. [12]	Mask R-CNN	Original 1741 images	Sensitivity = 0.96 False positives per image = 0.105	Dice index = 0.54
Teramoto et al. [13]	U-Net + CNN	Original 1741 images	Sensitivity = 0.98 False positives per image = 0.011	Dice index = 0.56
Proposed method	Cascade CNN	Original 2378 images	Sensitivity = 1.00 False positives per image = 0.005	Dice index = 0.56

## Data Availability

The source code and additional information used to support the findings of this study will be made available from the corresponding author upon request.
